# SphK1 Suppresses Human Dental Pulp Stem Cell Apoptosis by Promoting Glycolysis Under Simulated Microgravity

**DOI:** 10.1155/term/6659059

**Published:** 2025-12-16

**Authors:** Jingyi Che, Zhengjun Qiu, Huailong Hou, Yanping Li, Lina He, Jingxuan Sun, Shuang Zhang, Mengdi Li, Shuang Pan, Weiwei Zhang, Yumei Niu

**Affiliations:** ^1^ Department of Endodontics, First Affiliated Hospital of Harbin Medical University, Harbin, China, hrbmu.edu.cn; ^2^ Department of Endodontics, School of Stomatology, Harbin Medical University, Harbin, China, hrbmu.edu.cn

**Keywords:** apoptosis, glycolysis, human dental pulp stem cells, simulated microgravity, SphK1

## Abstract

Glycolysis supports mesenchymal stem cell (MSC) proliferation and sustains their undifferentiated state by maintaining energy supply and limiting apoptosis. The rapid advancement of space life sciences has spurred considerable interest in the effects of microgravity on stem cells. However, the contribution of glycolytic metabolism to apoptotic regulation under simulated microgravity (SMG) remains unclear. This study examined the influence of SMG on glycolytic activity and apoptosis in human dental pulp stem cells (hDPSCs). Lactic acid and glucose measurements were used to evaluate glycolytic flux, while transcript levels of HK2, PKM2, and LDHA were quantified by qPCR, HK2 and PKM2 protein expression was assessed by Western blotting, and annexin V‐FITC/PI staining combined with immunoblotting of apoptosis‐related proteins (BAX, BCL‐2, and cleaved caspase‐3) was performed to assess cell death. SMG markedly increased glycolytic capacity and attenuated apoptosis in hDPSCs. SphK1 expression was also elevated, indicating a role in cell survival. Pharmacological inhibition of SphK1 with PF‐543 reduced both glycolysis and the antiapoptotic effect, implicating SphK1 as a critical regulator of these processes. Inhibition of glycolysis by 2‐DG further increased apoptosis, confirming the protective role of glycolytic metabolism under SMG. These findings demonstrate that SMG enhances glycolysis and limits apoptosis in hDPSCs via SphK1 upregulation, suggesting that microgravity conditions may augment stem cell survival and function.

## 1. Introduction

Advances in tissue engineering and regenerative medicine depend on integrating cells, biomaterials, and signaling cues to restore damaged tissues. Classical approaches combine seed cells, scaffold structures, and bioactive molecules [1]. In recent years, stem cells have emerged as indispensable elements due to their proliferation, multipotency, and relatively low immunogenicity. As a subset of MSCs, human dental pulp stem cells (hDPSCs) can differentiate into odontoblasts, osteoblasts, adipocytes, vascular cells, and neurons, making them highly attractive for regenerative therapies [2, 3]. Progress in this field hinges on improving control over pluripotent stem cell behavior, differentiation, and culture conditions, with cell culture strategies remaining a cornerstone of regenerative research. Simulated microgravity (SMG) has gained attention as a model to examine the impact of altered gravitational forces on stem cell behavior, especially when combined with emerging technologies, such as three‐dimensional bioprinting and organ‐on‐a‐chip systems [4].

Microgravity triggers broad cellular adaptations, including changes in cytoskeletal structure, adhesion, motility, proliferation, differentiation, self‐renewal, and metabolism [5]. Previous work has shown that altered gravity affects tissue engineering efforts in multiple organ systems, including teeth, cartilage, bone, vasculature, heart, and liver, and offers opportunities for translational applications [6]. For instance, Elisabetta et al. cultured human bone marrow stem cells on nanocrystalline magnesium‐doped hydroxyapatite/type I collagen scaffolds (MHA/Coll) under SMG, showing that this scaffold environment enhanced osteogenic differentiation and reversed long‐term microgravity‐induced impairment. Such results suggest that microgravity can serve as a stringent testing platform for scaffold performance, advancing its translational potential in regenerative medicine [7]. Previous studies published by our team have also demonstrated that SMG enhanced the replicative activity, proliferation, and self‐renewal capacity of DPSCs, effectively slowing down their senescence process, while SMG was also able to elevate the multidirectional differentiation potential of DPSCs, with the upregulation of MSC surface markers and stemness maintenance‐related genes. [8], reinforcing the relevance of microgravity‐based models for tissue engineering research and prompting a wealth of ongoing research.

Cellular energy metabolism is central to stem cell function. Key metabolic pathways include aerobic oxidation, anaerobic glycolysis, and the pentose phosphate pathway, with glycolysis and mitochondrial oxidative phosphorylation (OXPHOS) playing dominant roles. Undifferentiated MSCs rely primarily on glycolysis to support rapid proliferation and self‐renewal, while differentiation induces a metabolic shift toward OXPHOS, accompanied by increased mitochondrial mass, upregulation of mitochondrial enzymes, reduced glycolytic activity, and enhanced oxygen consumption in the differentiated state [9–11]. Pharmacological stimulation of glycolysis using PS48 has also been shown to enhance hMSC self‐renewal, whereas inhibition of such metabolic activity using 2‐DG impairs proliferation [12]. Similarly, ALDOA knockdown in human spermatogonial stem cells reduces proliferation and glycolytic flux while increasing apoptosis [13]. Collectively, these findings highlight the role of glycolysis in sustaining stem cell viability, delaying differentiation, and suppressing apoptosis [12–14]. Apoptosis, although essential for normal development and tissue homeostasis, can be triggered by pathological conditions, leading to cellular dysfunction and tissue impairment [15, 16].

Sphingolipid‐related proteins constitute a major class of bioactive lipids involved in structural integrity and intracellular signaling. These molecules have been shown to regulate proliferation and survival across various progenitor cell types, including neural, muscle, hematopoietic, endothelial, and mesenchymal lineages [17]. Among them, sphingosine kinase 1 (SphK1) converts ceramide and sphingosine into sphingosine‐1‐phosphate (S1P), a metabolite implicated in nutrient signaling and vascular development [18]. The S1P1 axis governs key processes, such as embryonic angiogenesis, while SphK1‐driven upregulation of HIF‐1*α* enhances glycolytic adaptation under high‐altitude conditions [19]. Inhibition of SphK1 with PF‐543 has been shown to disrupt PFKFB3‐mediated glycolysis, thereby impairing tumor angiogenesis [20]. SphK1 also modulates cell fate by regulating the balance between the pro‐apoptotic protein Bax and the antiapoptotic protein BCL‐2. The combined actions of SphK1 and S1P regulate follicular cell proliferation, differentiation, and apoptosis through the PI3K/AKT/mTOR and ERK/MAPK signaling pathways [21]. Reduced SphK1 activity contributes to macrophage pyroptosis and tumor suppression [22]. Despite these insights, the role of SphK1 in controlling glycolysis and apoptosis in hDPSCs under SMG conditions remains undefined.

To address this gap in knowledge, this study was developed to investigate how SMG influences apoptosis, glycolytic metabolism, and SphK1 expression in hDPSCs, with the objective of clarifying how SphK1‐mediated glycolysis contributes to cell survival under SMG.

## 2. Materials and Methods

### 2.1. Isolation and Culture of hDPSCs

All procedures were approved by the Ethics Committee of the First Hospital of Harbin Medical University (IRB‐AF/SC‐04/02.0) and conducted in accordance with institutional guidelines. Healthy permanent teeth were obtained from adults aged 16–25 undergoing extraction for orthodontic or impaction reasons. Under aseptic conditions in a laminar flow hood, each tooth was rinsed twice in phosphate‐buffered saline (PBS; Servicebio, Wuhan, China) supplemented with penicillin (100 U/mL) and streptomycin (100 μg/mL) (Seven Biotech, Beijing, China). Pulp tissue was gently removed with a pulp needle, trimmed to eliminate root portions, and washed three times in Dulbecco’s Modified Eagle Medium (DMEM; Gibco, Grand Island, CA, USA). The pulp was minced into fragments of approximately 1 mm^3^ in DMEM and transferred to 15‐mL centrifuge tubes (Servicebio, Wuhan, China). Enzymatic digestion was performed with type I collagenase (Boster, China) and dispase (Sigma, Saint Louis, MO, USA). After neutralization, cell suspensions were centrifuged at 1500 rpm for 5 min, supernatants discarded, and pellets resuspended in DMEM containing 20% fetal bovine serum (Excell, China). Cells were seeded evenly into T25 flasks (Corning, USA) and maintained at 37°C in a 5% CO_2_ incubator (Thermo, USA). Medium was refreshed after 3 days to remove nonadherent cells and subsequently replaced every 2 days until cultures reached 70%–80% confluence for passaging. Cells at passages 3–7 were used for all experiments.

### 2.2. Evaluation of hDPSC Differentiation Potential

#### 2.2.1. Alizarin Red Staining

Cells were maintained in osteogenic induction medium (Oricell, Guangzhou, China) for 21 days and then fixed in 4% paraformaldehyde for 20 min. After three PBS washes, 0.5 mL of Alizarin Red solution (Beyotime, Shanghai, China) was added to each well and incubated at room temperature for 5–10 min. Excess stain was removed, cells were rinsed three times with PBS, and mineralized nodules were visualized and photographed under an optical microscope.

#### 2.2.2. Chondrogenic Differentiation

Cells were plated and grown for 2 days until > 90% confluence and then switched to chondrogenic induction medium (Oricell, Guangzhou, China). Medium was changed every 2 days for 21 days, and cell morphology was documented microscopically.

#### 2.2.3. Adipogenic Differentiation

Cells were seeded and cultured for 2 days until reaching > 70% confluence and then transferred to adipogenic induction medium (Oricell, Guangzhou, China). Medium changes occurred every 2 days. After 21 days, cells were examined and photographed under a microscope.

### 2.3. SMG Induction

Ground‐based microgravity conditions were produced using the CellSpace‐3D tri‐axial culture device (CellSpace, Beijing, China), which utilizes a 3D inclinometer with two rotational axes and applies 45° rotation about these dual axes to disperse the gravity vector and thereby simulate microgravity. T25 flasks were randomly assigned to either the SMG or the static control groups. Following 24 h of adhesion, when cells had reached ∼80% confluence, flasks were completely filled with medium to minimize fluid shear stress while carefully ensuring the removal of all bubbles. SMG flasks were mounted on the clinostat and rotated at 4 rpm within a 37°C and 5% CO_2_ incubator for 72 h. Control flasks remained stationary under identical incubator conditions.

### 2.4. Flow Cytometry

Surface marker expression of hDPSCs was evaluated by flow cytometry. A total of 5 × 10^5^ cells (passage 3) were harvested, washed in PBS, and incubated at 4°C in the dark with antibodies against human CD90, CD105, CD34, and CD45 (Thermo Fisher Scientific, Waltham, MA, USA). Unstained cells served as negative controls. After 30 min, cells were washed three times with PBS, resuspended in 300 μL PBS, and analyzed using a BD LSRII flow cytometer (BD Biosciences).

### 2.5. Western Blotting

Cells were lysed on ice with RIPA buffer (Beyotime, Shanghai, China) containing protease inhibitor PMSF (Beyotime, Shanghai, China) at 100:1. Lysates were sonicated and centrifuged at 13,500 rpm for 20 min, and supernatants were collected. Proteins were denatured in SDS–PAGE loading buffer (Beyotime, Shanghai, China) at 100°C for 10 min, separated on 10% SDS–PAGE gels, and transferred to PVDF membranes (Millipore, Billerica, MA, USA). Membranes were blocked in 5% skim milk/TBST for 1 h at room temperature and incubated overnight at 4°C with primary antibodies against HK2, PKM2, BAX, BCL‐2, caspase‐3 (Proteintech, Wuhan, China), SphK1 (MCE, China), and β‐actin (ABclonal, Wuhan, China). After three TBST washes, HRP‐conjugated secondary antibodies (ZSGB‐BIO, Beijing, China) were applied for 1 h at room temperature. Protein bands were visualized using an enhanced chemiluminescence (ECL) detection system.

### 2.6. qPCR

Total RNA was extracted using the RNA isolation kit (Goonie Bio, China). RNA concentrations were determined with a NanoDrop 2000 spectrophotometer (Thermo Fisher Scientific, Waltham, MA, USA). One microgram of RNA was reverse‐transcribed to cDNA using the Prime RT Reagent Kit (TaKaRa, Kusatsu, Japan). qPCR was performed on a QuantStudio 6 Real‐Time PCR System (Thermo, USA) using SYBR Green chemistry (Seven Biotech, Beijing, China). The cycling parameters were 95°C for 30 s followed by 40 cycles of 95°C for 10 s and 60°C for 20 s. Expression of target genes was normalized to β‐actin. Primer sequences were as follows: β‐ACTIN F‐GCACTCTTCCAGCC TTCCTTCCTG, R‐GGAGTACTTGCGCTCAGGAGGAGC; human HK2 F‐TCTA CCACATGCGCCTCTCT, R‐GCCCATTGTCCGTTACTTTC; human PKM2 F‐CCA CTT​GCA​ATT​ATT​TGA​GGA​A, R‐GTGAGCAGACCTGCCAGACT; human LDHA F‐TGGAGTGGAATGAATGTTGC, R‐ATAGCCCAGGATGTGTAGCC; human SphK1 F‐CGCTTCACTCTGGGCACCTTC, R‐TCGTCGGG CACCACTGTCC.

### 2.7. Glucose Consumption Assay

Glucose uptake from the culture medium was quantified using the glucose oxidase assay (Jiancheng, Nanjing, China). Cells were seeded into 96‐well plates and maintained at 37°C in a humidified incubator (5% CO_2_) for 24 h following sample addition. After incubation, supernatants were collected from each well, and glucose concentrations were determined according to the manufacturer’s instructions. Glucose consumption in each experimental group was calculated by subtracting the remaining glucose concentration from the initial concentration.

### 2.8. Lactate Production Assay

Cellular lactate levels were assessed with a lactate content kit (Jiancheng, Nanjing, China). hDPSCs were harvested, centrifuged at 1000 rpm, and subjected to ultrasonic disruption in PBS. Total protein concentrations were determined using the BCA method at 562 nm, and lactate production was normalized to protein content in accordance with the kit protocol.

### 2.9. Mitochondrial Membrane Potential Analysis

To evaluate mitochondrial membrane potential, 6 × 10^5^ cells were plated per 35 mm dish and allowed to adhere overnight at 37°C in 5% CO_2_. Cells were then resuspended in 500 μL of fresh culture medium and gently mixed with 500 μL of JC‐1 working solution. Following 20‐min incubation at 37°C, cells were centrifuged at 600 g for 4 min at 4°C, supernatants discarded, and pellets washed twice with JC‐1 buffer under identical centrifugation conditions. The final pellet was resuspended in 500 μL PBS, kept on ice, and protected from light until analysis. Green fluorescence (monomeric JC‐1) was detected in the FITC channel and red fluorescence (aggregated JC‐1) in the PE channel using a flow cytometer. Median fluorescence intensities (MFI) for each channel were recorded, and the red/green MFI ratio was calculated to determine mitochondrial membrane potential.

### 2.10. Statistical Analysis

All data were normalized to the control group and expressed as mean ± S.E.M. Each assay was repeated a minimum of three times. Two‐tailed Student′s *t*‐tests were used to evaluate differences between two groups. A *p*‐value < 0.05 was considered significant.

## 3. Results

### 3.1. Isolation, Culture, and Identification of hDPSCs

Enzymatic digestion of pulp tissue yielded hDPSCs that migrated outward from tissue fragments within 3–7 days, displaying a spindle‐like morphology. Confluence was typically reached by days 7–14, at which point cells formed extensive intercellular contacts and were subcultured (Figure 1(a)). After 21 days of osteogenic induction, Alizarin Red staining revealed robust mineralized nodule formation (Figure 1(b)). Adipogenic induction for 21 days produced visible lipid droplets (Figure 1(c)), while chondrogenic differentiation resulted in cartilage‐like nodules (Figure 1(d)). Flow cytometry confirmed mesenchymal identity, with strong CD90 and CD105 expression and negligible CD34 and CD45 staining at passage three (Figure 1(e)).

Figure 1Isolation, culture, and identification of DPSCs. (a) After 14 days of extracting human pulp tissue, it showed fusion and connection; (b) DPSCs stained with Alizarin Red; (c) adipogenic differentiation of hDPSCs; (d) chondrogenic differentiation of hDPSCs; (e) the expression of CD34, CD45, CD90, and CD105 of hDPSCs.

**Figure:**
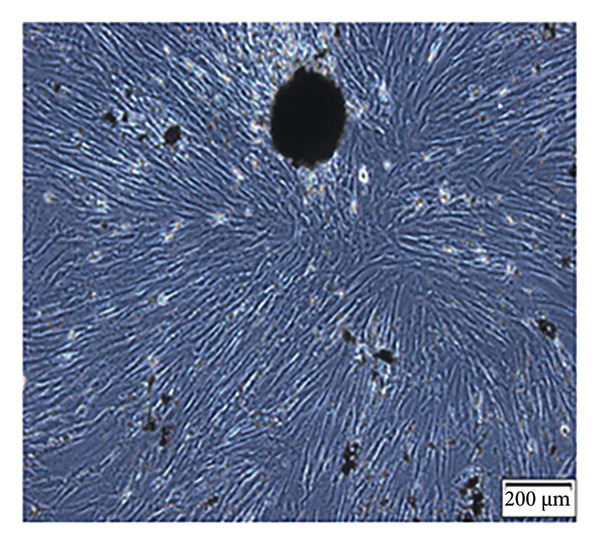
(a)

**Figure:**
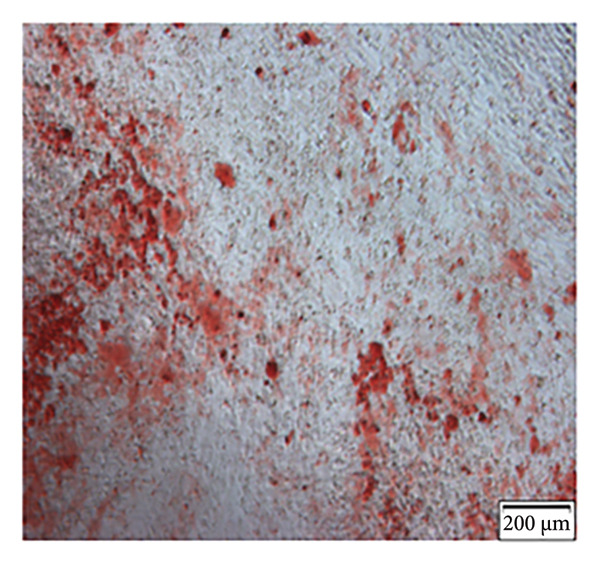
(b)

**Figure:**
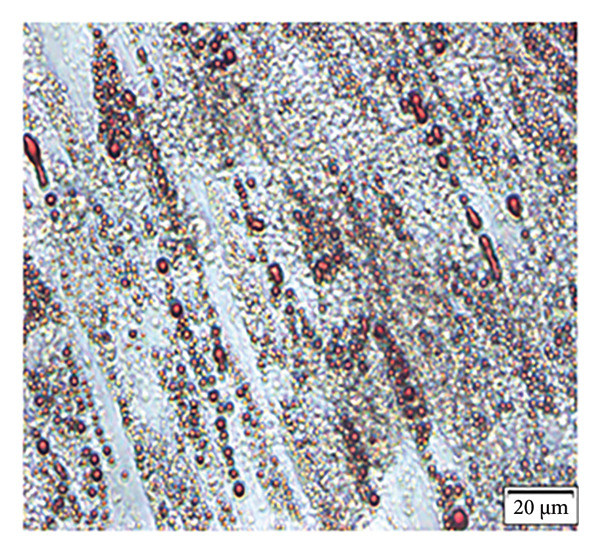
(c)

**Figure:**
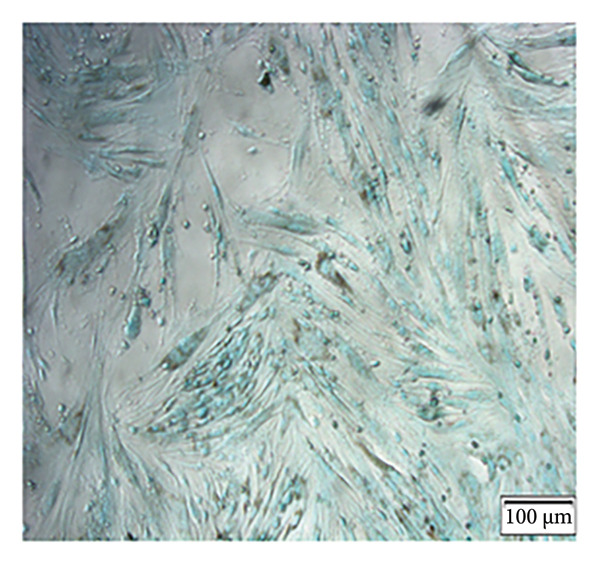
(d)

**Figure:**
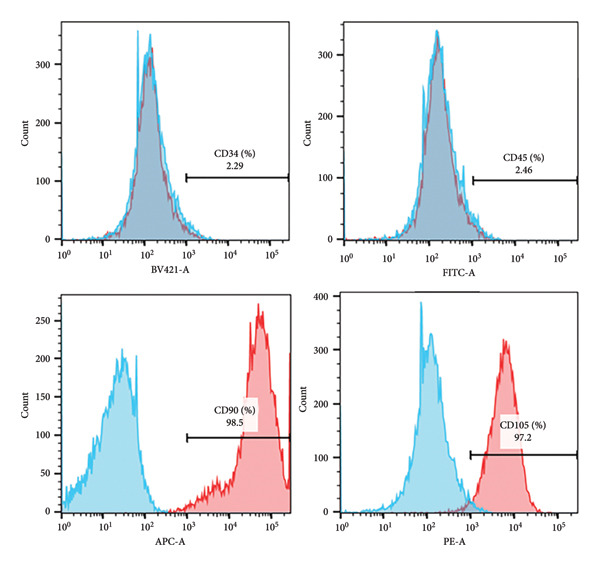
(e)

### 3.2. Effects of SMG Conditions on hDPSC Apoptosis

Western blotting revealed decreased levels of pro‐apoptotic BAX, increased antiapoptotic BCL‐2, a reduced BAX/BCL‐2 ratio, and diminished cleaved caspase‐3 activation in SMG‐treated cells (Figures 2(a), 2(b), 2(c), 2(d), 2(e)), indicating that SMG suppresses apoptotic activity in hDPSCs. Flow cytometric analysis indicated that apoptosis was markedly reduced under SMG relative to normal gravity (NG) conditions (Figures 2(f), 2(g)).

Figure 2Effects of SMG on the apoptosis of hDPSCs. (a) BAX, BCL2, and cleaved caspase‐3 protein blot images; (b) quantitative analysis of relative expression of BAX protein (^∗^ *p* < 0.05, *n* = 3); (c) quantitative analysis of relative expression of BCL2 protein (^∗∗^ *p* < 0.01, *n* = 3); (d) quantitative analysis of relative expression of BAX/BCL2 protein (^∗∗^ *p* < 0.01, *n* = 3); (e) quantitative analysis of relative expression of cleaved caspase‐3 protein (^∗^ *p* < 0.05, *n* = 3); (f) JC‐1 assay for mitochondrial membrane potential detection analyzed by flow cytometry; (g) JC‐1 red/green ratio analysis bar chart. (^∗^ *p* < 0.05, *n* = 3).

**Figure:**
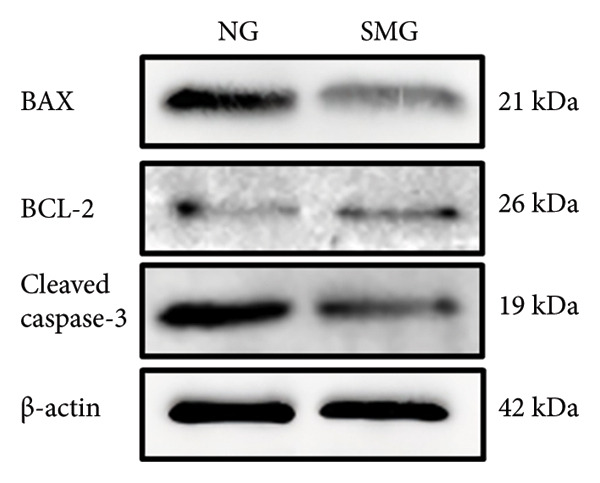
(a)

**Figure:**
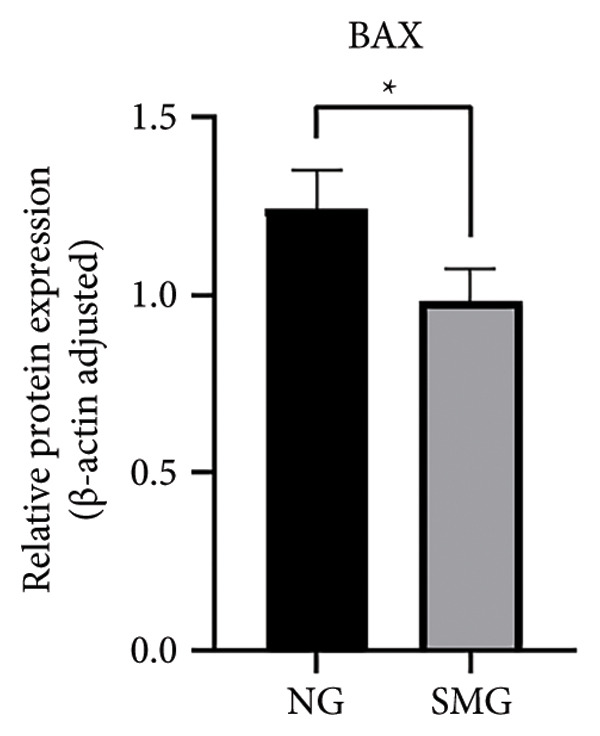
(b)

**Figure:**
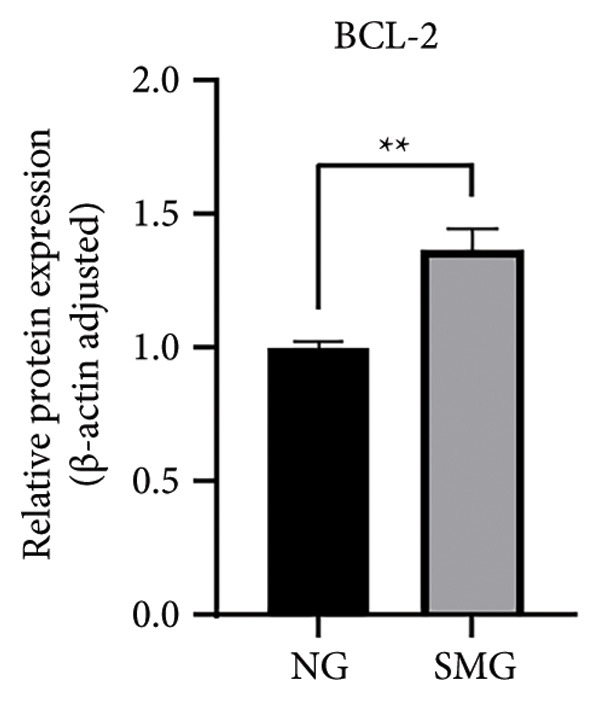
(c)

**Figure:**
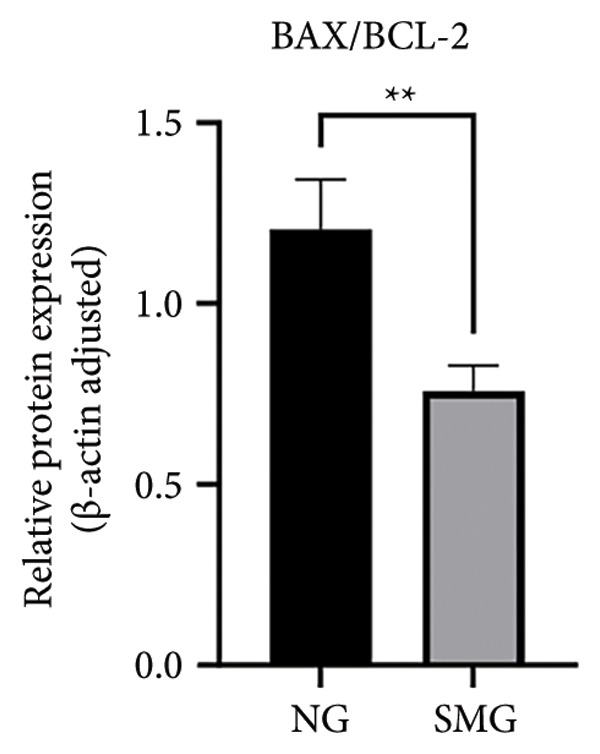
(d)

**Figure:**
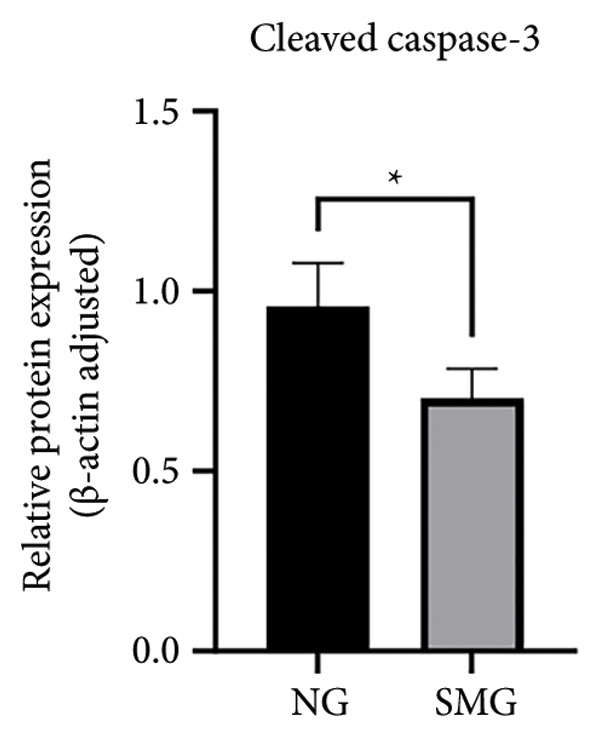
(e)

**Figure:**
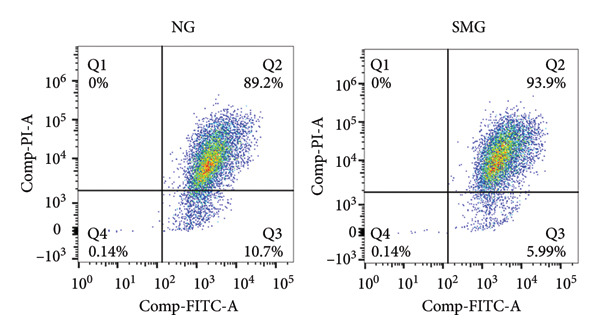
(f)

**Figure:**
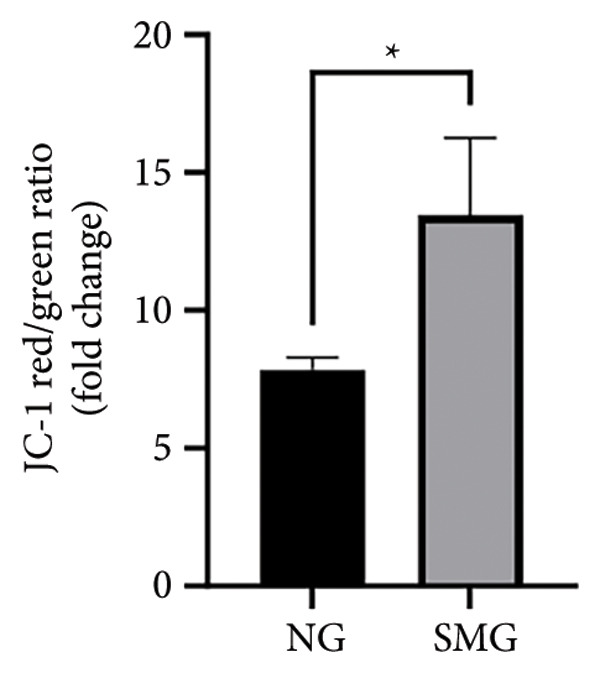
(g)

### 3.3. Effects of SMG on hDPSC Glycolytic Activity

Glucose consumption assays showed significantly increased uptake in SMG‐exposed cells compared with NG controls (Figure 3(a)). Consistently, lactate production assays demonstrated elevated lactate accumulation in the SMG group (Figure 3(b)). QPCR confirmed increased mRNA expression of HK2, PKM2, and LDHA under SMG conditions (Figures 3(c), 3(d), 3(e)), while Western blotting revealed upregulation of glycolytic enzymes HK2 and PKM2 (Figures 3(f), 3(g), 3(h)). Together, these findings indicate that SMG enhances glycolytic metabolism in hDPSCs.

Figure 3Effects of SMG on the glycolysis of hDPSCs. (a) Glucose consumption under SMG of hDPSCs for 3 days (^∗∗^ *p* < 0.01, *n* = 3); (b) lactic acid production under SMG of hDPSCs for 3 days (^∗∗^ *p* < 0.01, *n* = 3); (c–e): the mRNA expression of HK2 (^∗^ *p* < 0.05, *n* = 3); the mRNA expression of PKM2 (^∗∗^ *p* < 0.01, *n* = 3); the mRNA expression of LDHA. (^∗^ *p* < 0.05, *n* = 3). (f) HK2 and PKM2 protein blot images; (g) quantitative analysis of relative expression of HK2 protein (^∗^ *p* < 0.05, *n* = 3); (h) quantitative analysis of relative expression of PKM2 protein (^∗^ *p* < 0.05, *n* = 3).

**Figure:**
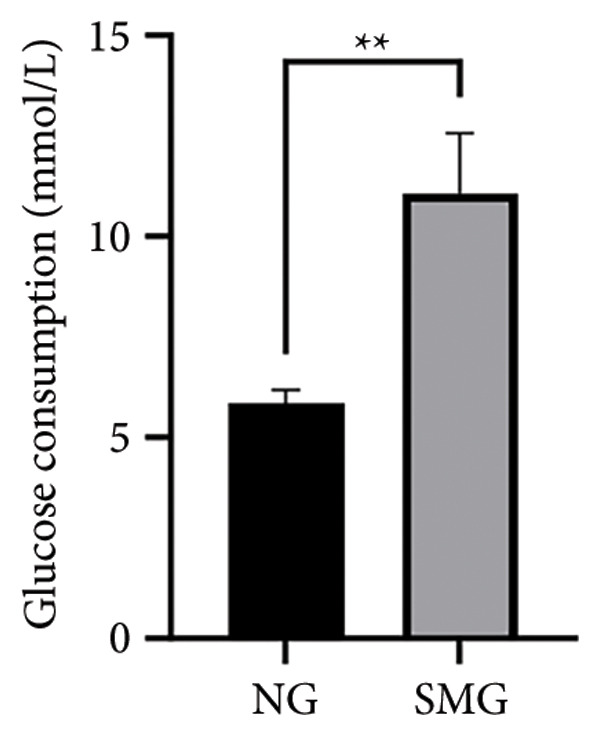
(a)

**Figure:**
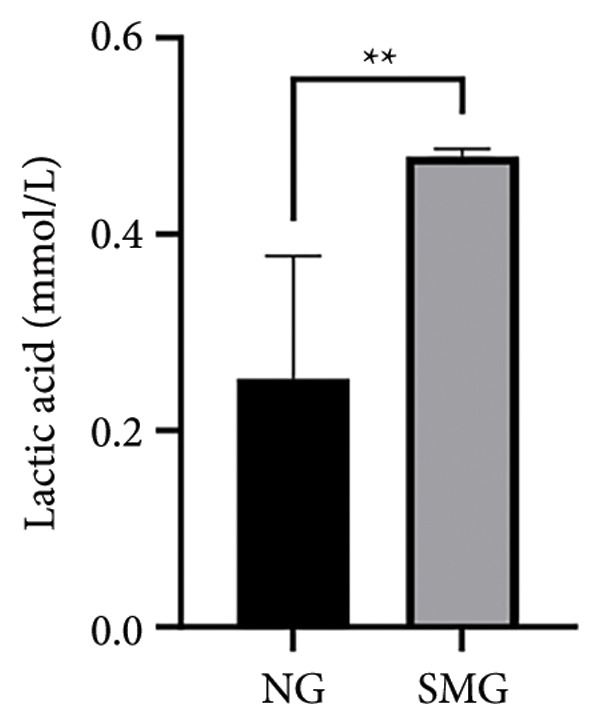
(b)

**Figure:**
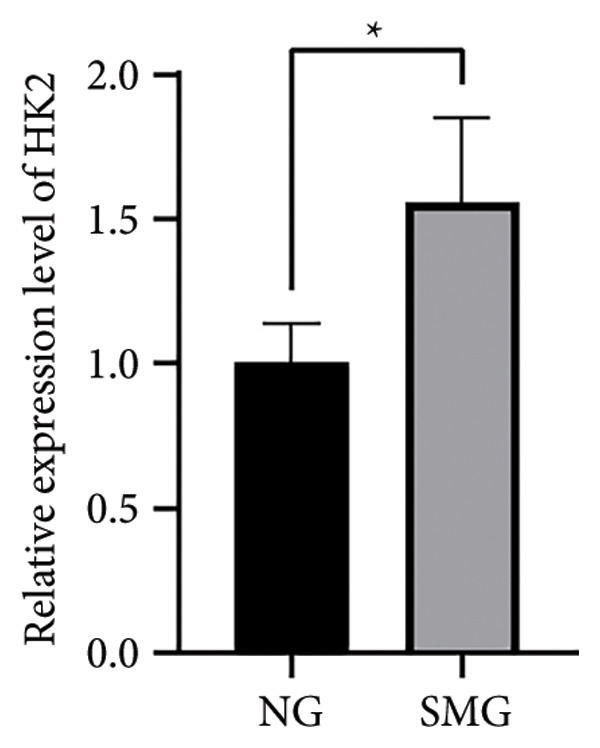
(c)

**Figure:**
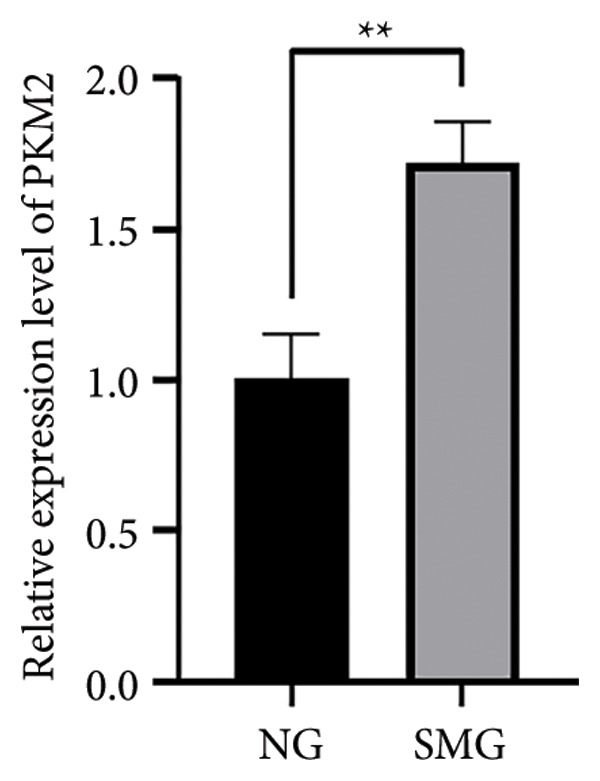
(d)

**Figure:**
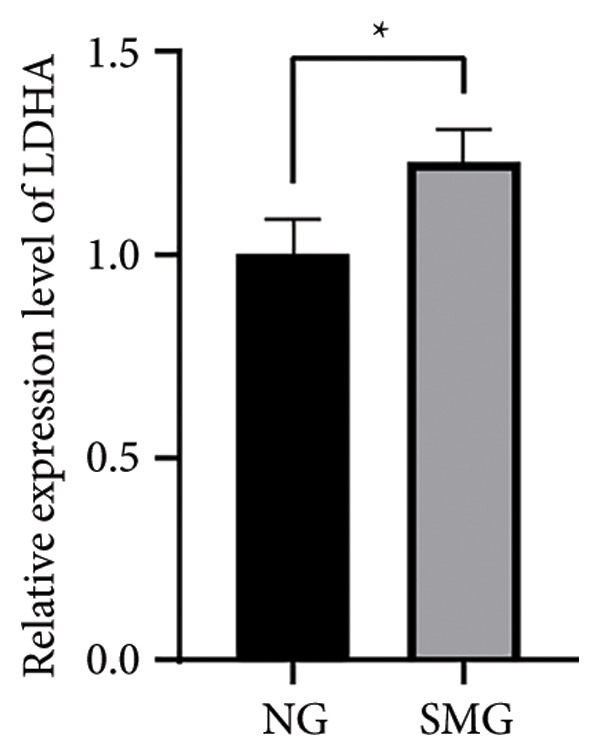
(e)

**Figure:**
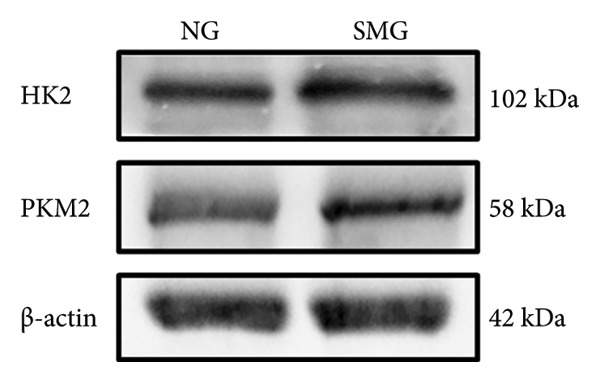
(f)

**Figure:**
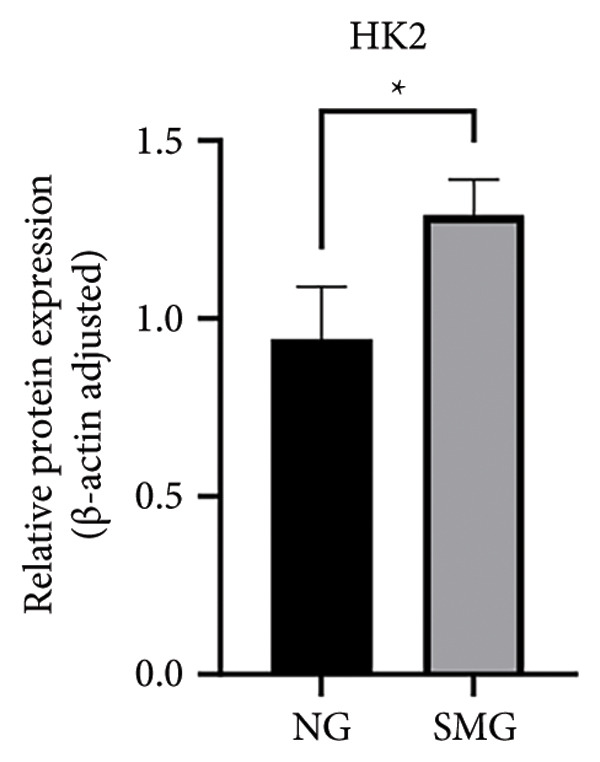
(g)

**Figure:**
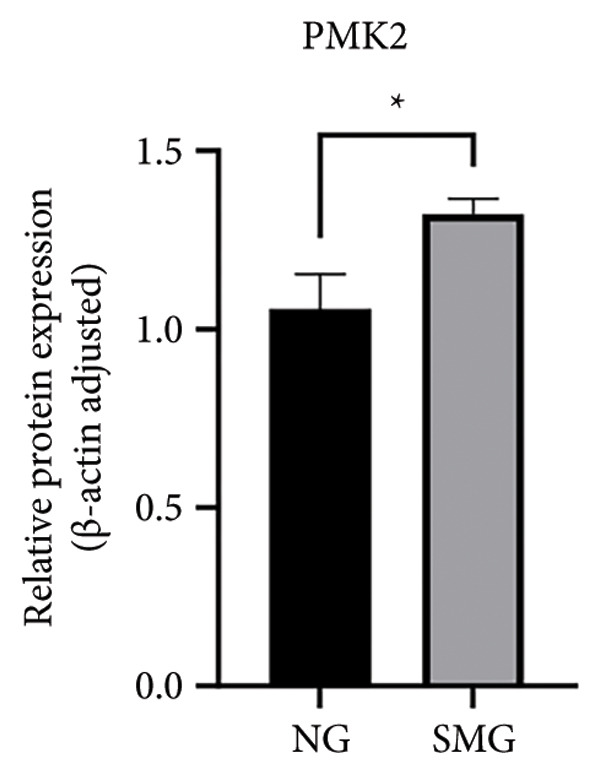
(h)

### 3.4. Impact of Glycolysis Inhibition on hDPSC Apoptosis

Western blotting showed increased BAX expression, decreased BCL‐2, a higher BAX/BCL‐2 ratio, and enhanced cleaved caspase‐3 (Figures 4(a), 4(b), 4(c), 4(d), 4(e)). Exposure to the glycolytic inhibitor 2‐DG (10 μM) increased apoptosis in SMG‐cultured hDPSCs. Flow cytometry revealed a higher apoptotic fraction (Figures 4(f), 4(g)). These results demonstrate that glycolytic activity contributes to the antiapoptotic effect of SMG in hDPSCs.

Figure 4Effects of glycolysis inhibitor 2‐DG on apoptosis of hDPSCs. (a) BAX, BCL2, and cleaved caspase‐3 protein blot images; (b) quantitative analysis of relative expression of BAX protein (^∗∗^ *p* < 0.01, *n* = 3); (c) quantitative analysis of relative expression of BCL2 protein (^∗∗∗^ *p* < 0.001, *n* = 3); (d) quantitative analysis of relative expression of BAX/BCL2 protein (^∗∗^ *p* < 0.01, *n* = 3); (e) quantitative analysis of relative expression of cleaved caspase‐3 protein (^∗^ *p* < 0.05, *n* = 3); (f) JC‐1 assay for mitochondrial membrane potential detection analyzed by flow cytometry; (g) JC‐1 red/green ratio analysis bar chart. (^∗^ *p* < 0.05, *n* = 3).

**Figure:**
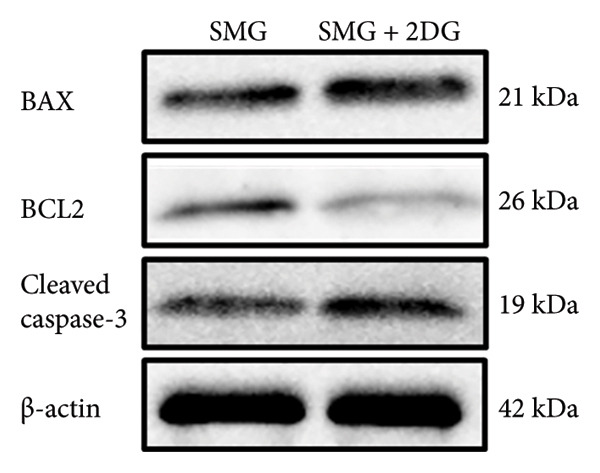
(a)

**Figure:**
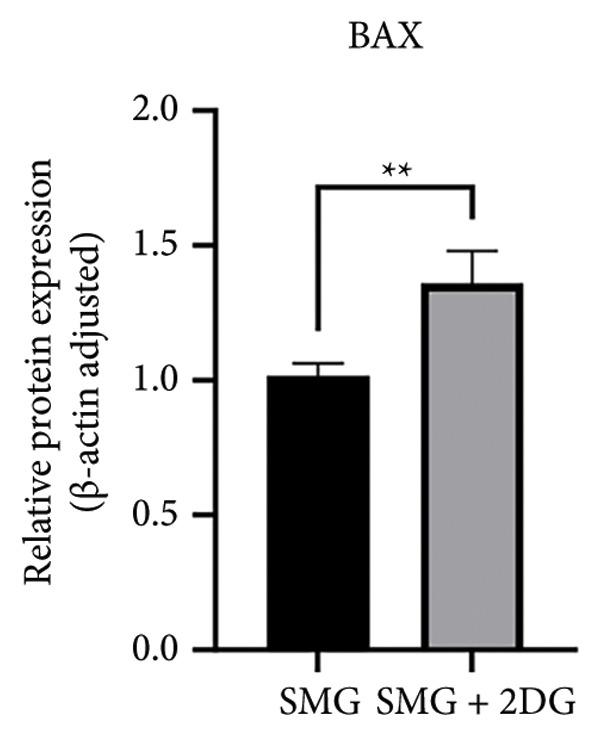
(b)

**Figure:**
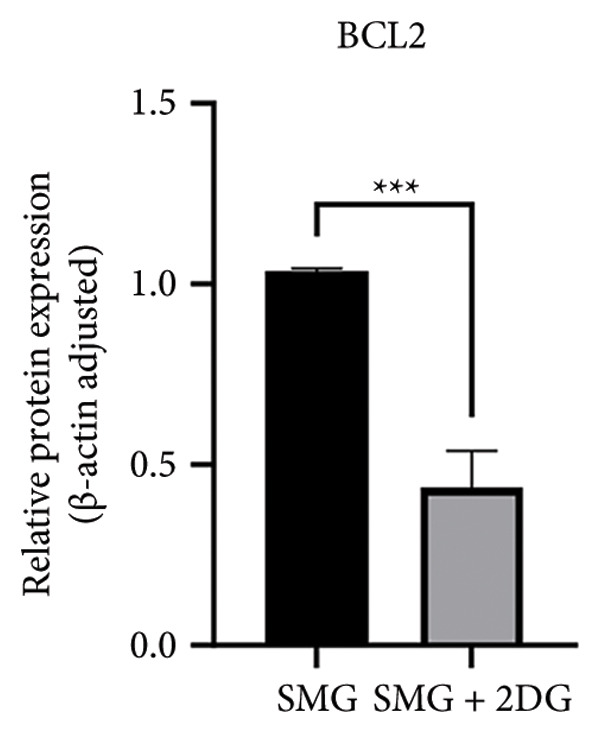
(c)

**Figure:**
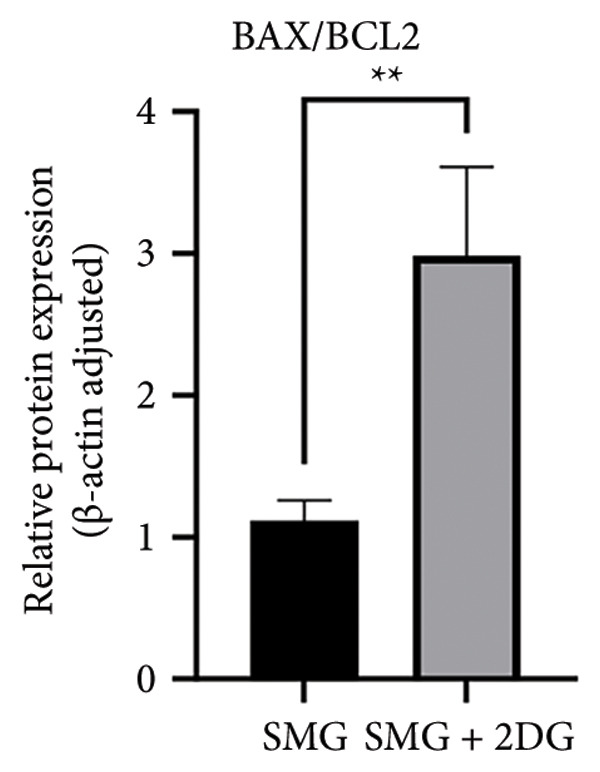
(d)

**Figure:**
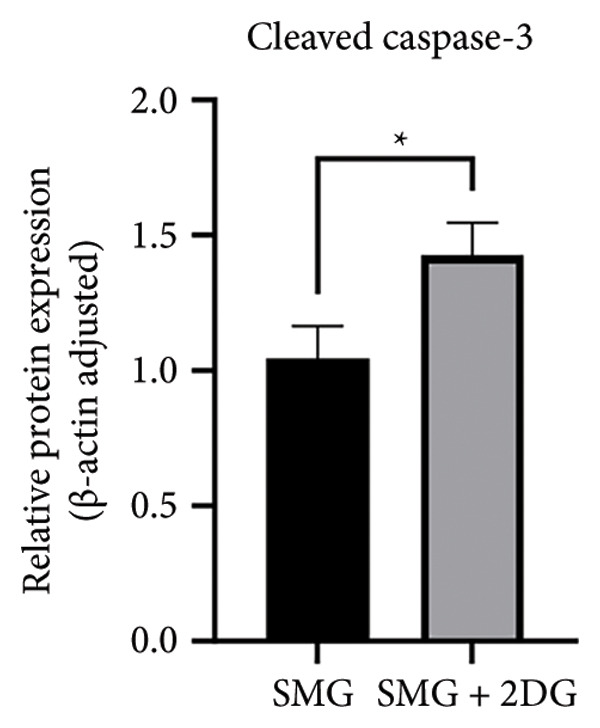
(e)

**Figure:**
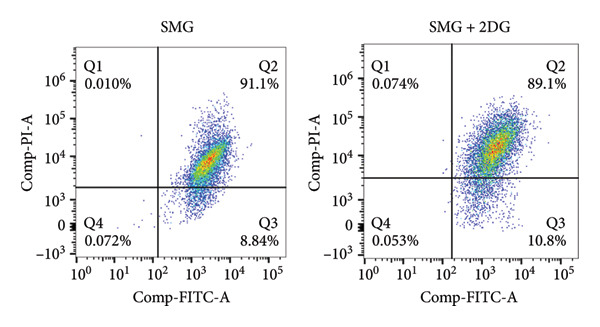
(f)

**Figure:**
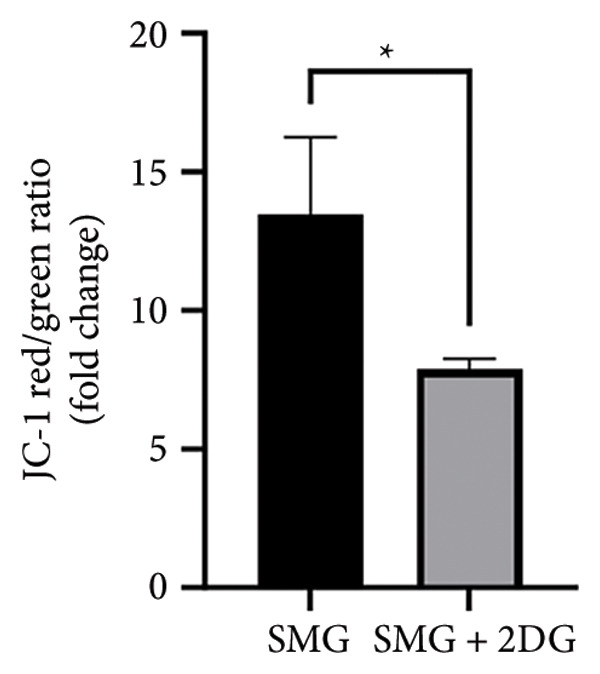
(g)

### 3.5. SphK1 Expression is Upregulated Under SMG

Proteomic and transcriptomic results revealed a significant increase in SphK1 (Figures 5(a), 5(b)). SphK1 expression at both mRNA and protein levels was significantly elevated in hDPSCs cultured under SMG compared with NG controls (Figures 5(c), 5(d), 5(e)). These findings suggest that SMG induces SphK1 upregulation.

Figure 5The expression of SphK1 under SMG of hDPSCs for 3 days. (a) Volcano plot of differential gene expression; (b) the expression of SphK1 in proteomic sequencing; (c) the mRNA expression of SphK1 (^∗^ *p* < 0.05, *n* = 3); (d) SphK1 protein blot images; (e) quantitative analysis of relative expression of SphK1 protein. (^∗^ *p* < 0.05, *n* = 3).

**Figure:**
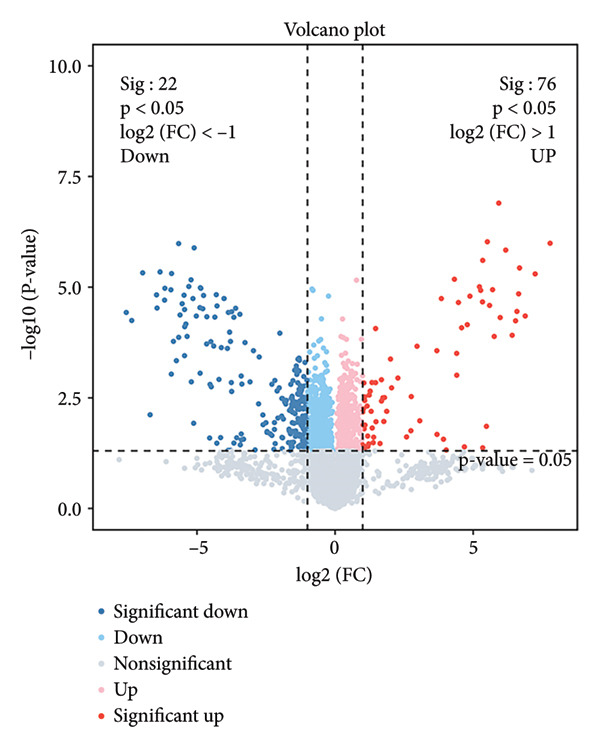
(a)

**Figure:**
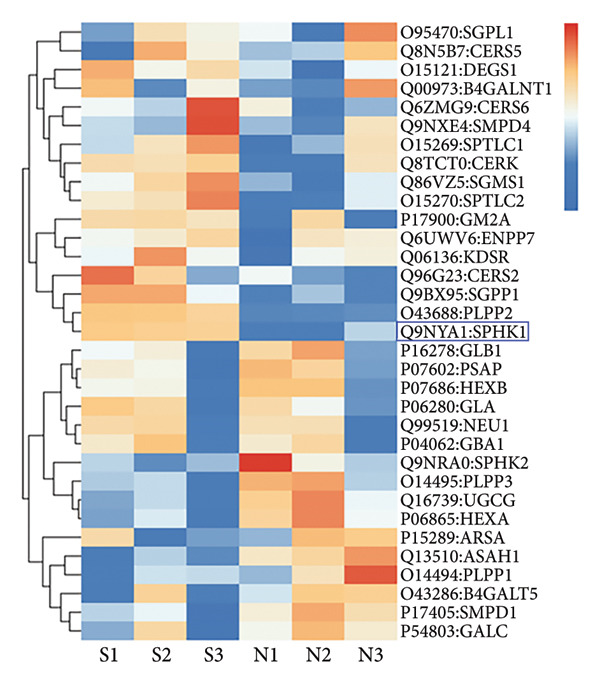
(b)

**Figure:**
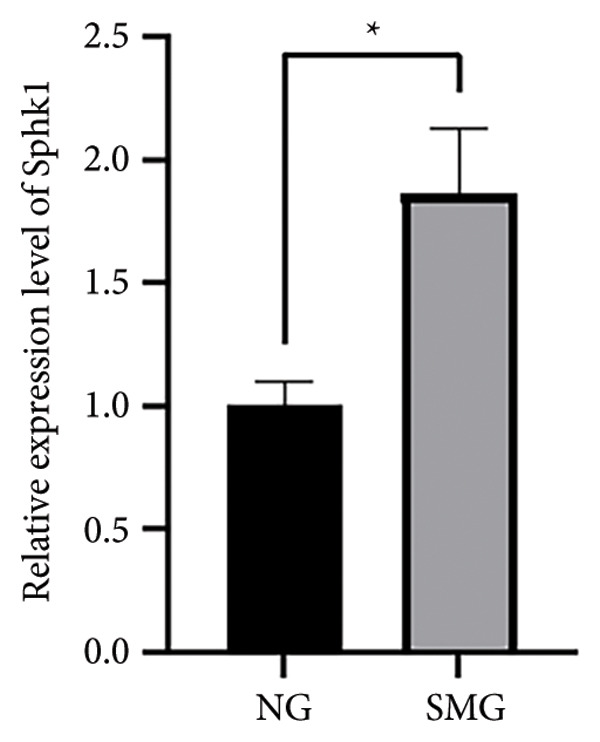
(c)

**Figure:**
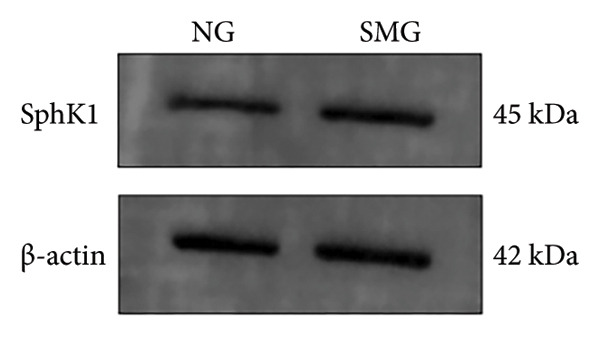
(d)

**Figure:**
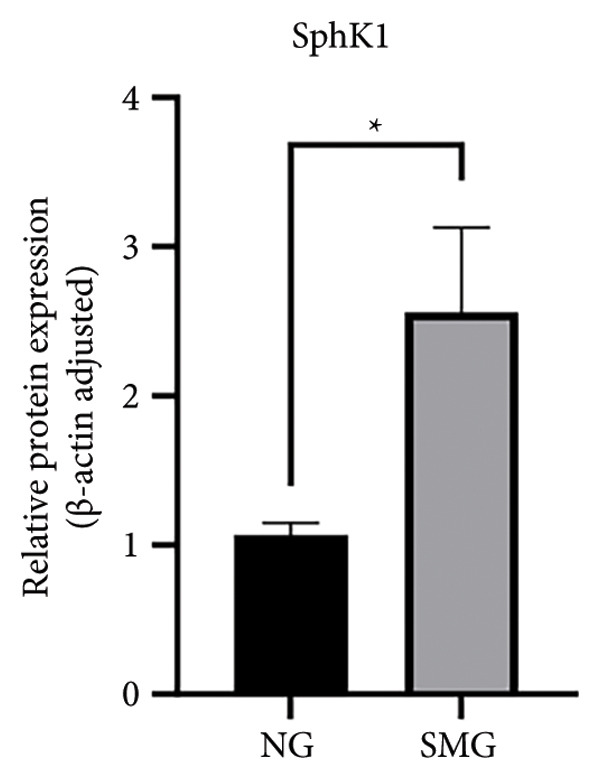
(e)

### 3.6. SphK1 Inhibition Modulates the Glycolytic Capacity of hDPSCs

Treatment with the SphK1 inhibitor PF‐543 (10 μM) under SMG conditions markedly reduced glucose uptake and lactate production compared with SMG alone (Figures 6(a), 6(b)). Western blotting demonstrated downregulation of HK2 and PKM2 (Figures 6(c), 6(d), 6(e)), and qPCR showed decreased expression of HK2, PKM2, and LDHA (Figures 6(f), 6(g), 6(h)). These findings indicate that SphK1 upregulation mediates SMG‐enhanced glycolysis in hDPSCs.

Figure 6Effects of SphK1 inhibitor PF‐543 on glycolytic capacity of hDPSCs. (a) Glucose consumption after using PF‐543 under SMG of hDPSCs for 3 days (^∗∗^ *p* < 0.01, *n* = 3); (b) lactic acid production after using PF‐543 under SMG of hDPSCs for 3 days (^∗∗^ *p* < 0.01, *n* = 3); (c) HK2 and PKM2 protein blot images; (d) quantitative analysis of relative expression of HK2 protein (^∗^ *p* < 0.05, *n* = 3); (e) quantitative analysis of relative expression of PKM2 protein (^∗^ *p* < 0.01, *n* = 3); (f‐h) the mRNA expression of HK2 (^∗∗^ *p* < 0.01, *n* = 3); the mRNA expression of PKM2 (^∗^ *p* < 0.05, *n* = 3); the mRNA expression of LDHA. (^∗∗^ *p* < 0.01, *n* = 3).

**Figure:**
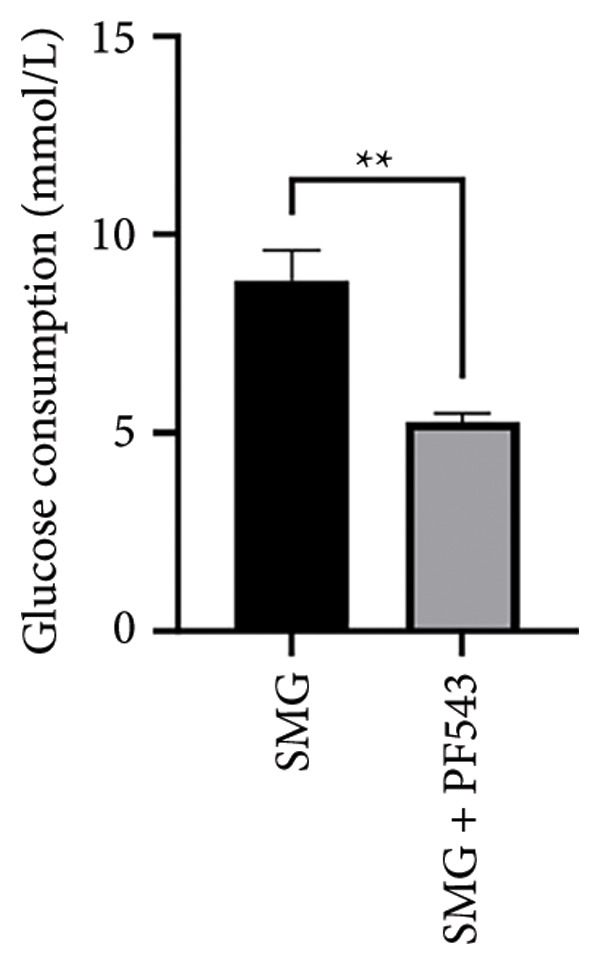
(a)

**Figure:**
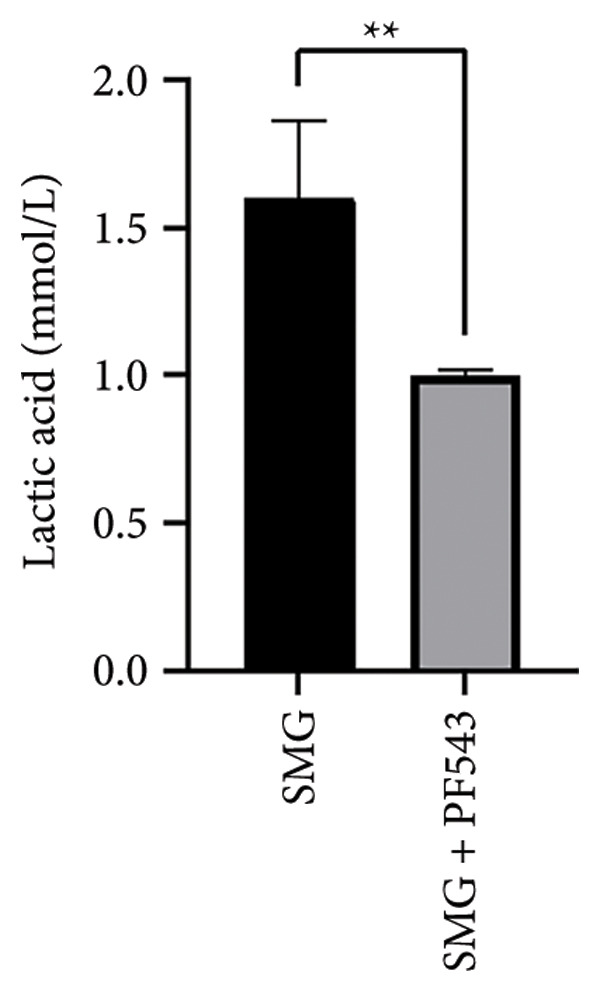
(b)

**Figure:**
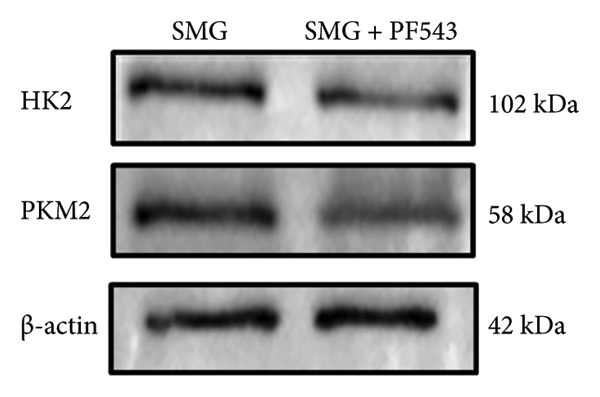
(c)

**Figure:**
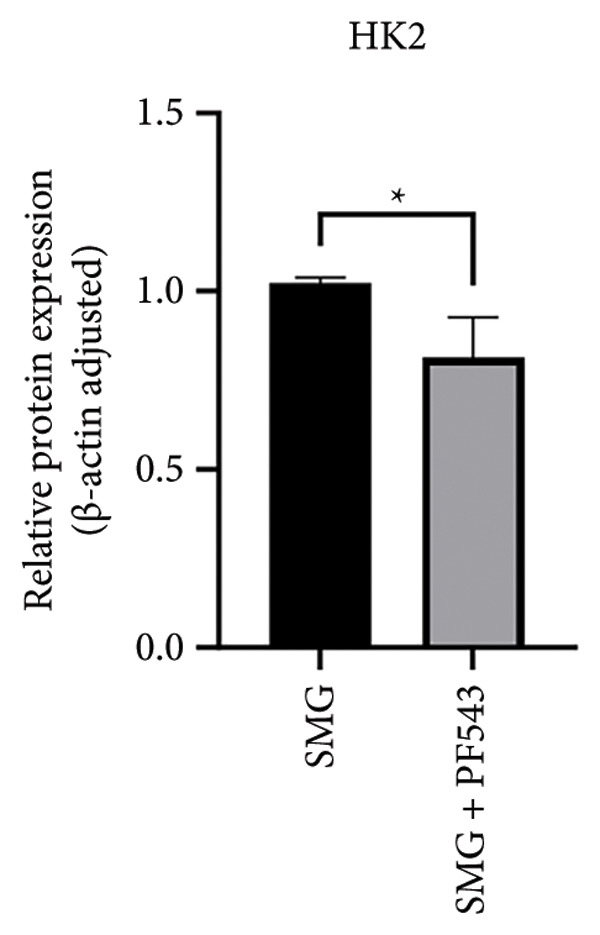
(d)

**Figure:**
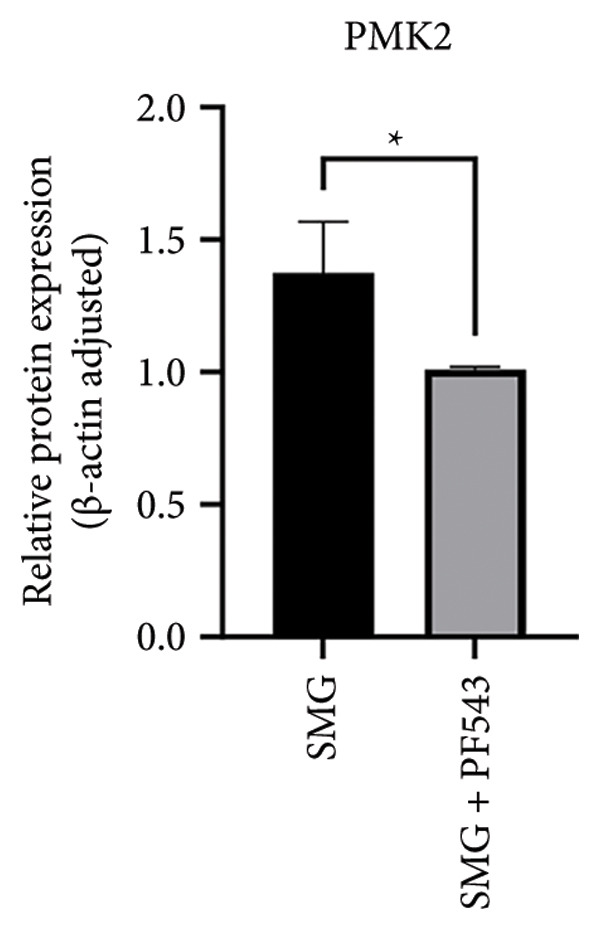
(e)

**Figure:**
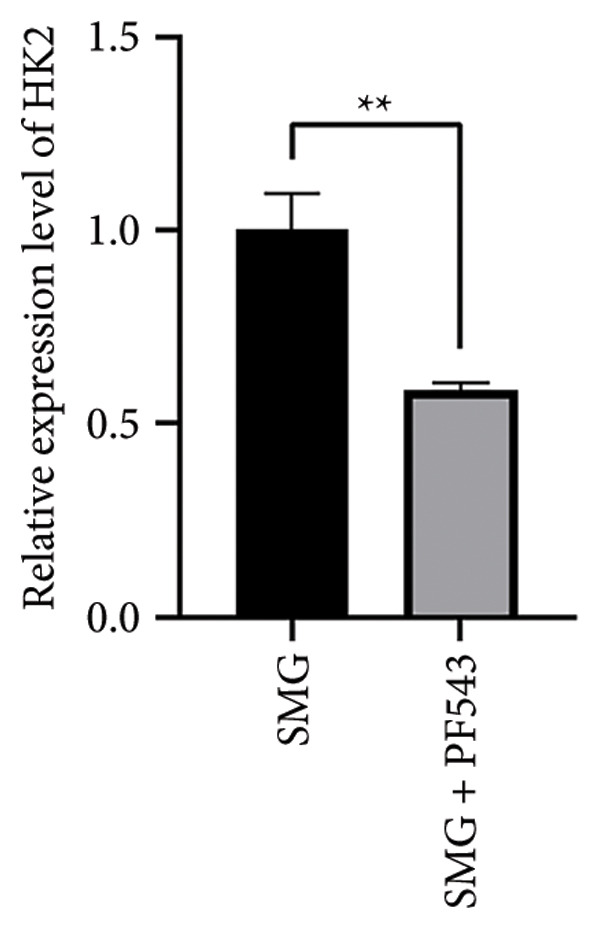
(f)

**Figure:**
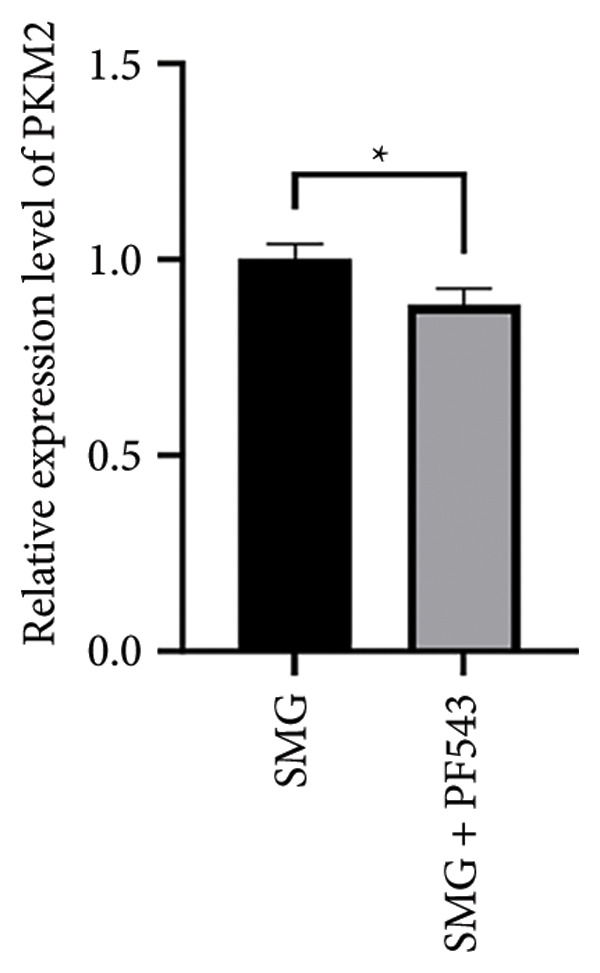
(g)

**Figure:**
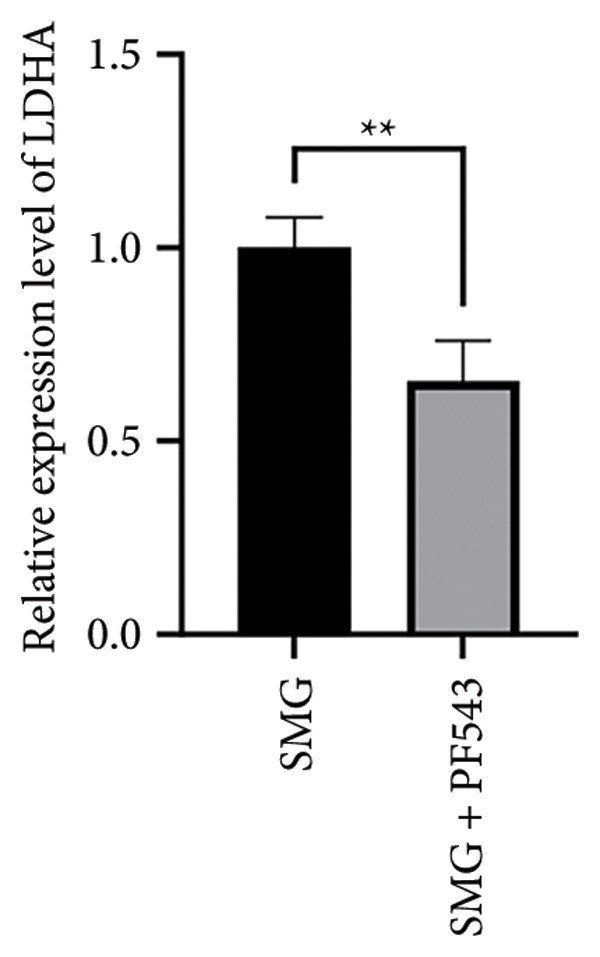
(h)

### 3.7. SphK1 Inhibition Modulates hDPSC Apoptosis

Lastly, the effects of PF‐543‐mediated SphK1 inhibition on hDPSC apoptosis were evaluated. Western blotting confirmed increased BAX, reduced BCL‐2, and enhanced cleaved caspase‐3 in the presence of PF‐543 (Figures 7(a), 7(b), 7(c), 7(d), 7(e)). Flow cytometry revealed a significant increase in apoptotic cells in the SMG + PF‐543 group compared with SMG alone (Figures 7(f), 7(g)). These data demonstrate that SphK1 activity contributes to the antiapoptotic effects of SMG in hDPSCs.

Figure 7Effects of SphK1 inhibitor PF‐543 on apoptosis of hDPSCs. (a) BAX, BCL2, and cleaved caspase‐3 protein blot images; (b) quantitative analysis of relative expression of BAX protein (^∗^ *p* < 0.05, *n* = 3); (c) quantitative analysis of relative expression of BCL2 protein (^∗^ *p* < 0.05, *n* = 3); (d) quantitative analysis of relative expression of BAX/BCL2 protein (^∗∗^ *p* < 0.01, *n* = 3); (e) quantitative analysis of relative expression of cleaved caspase‐3 protein (^∗^ *p* < 0.05, *n* = 3); (f) JC‐1 assay for mitochondrial membrane potential detection analyzed by flow cytometry; (g) JC‐1 red/green ratio analysis bar chart. (^∗∗^ *p* < 0.01, *n* = 3).

**Figure:**
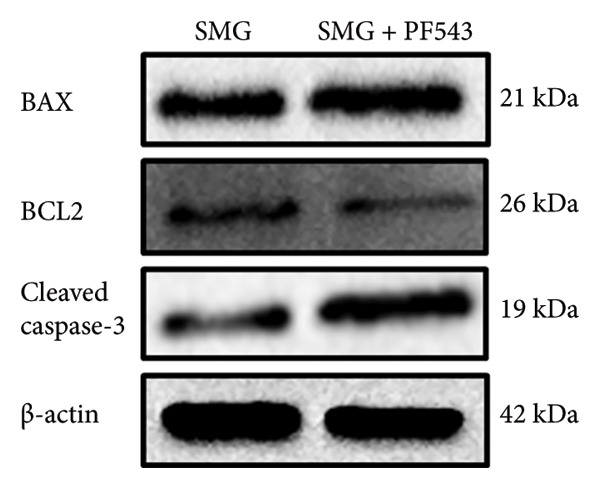
(a)

**Figure:**
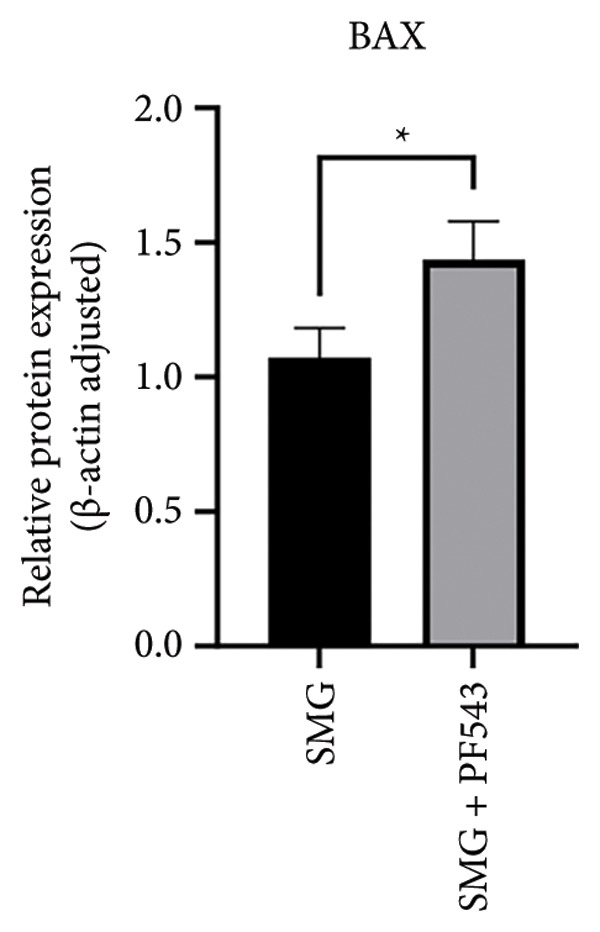
(b)

**Figure:**
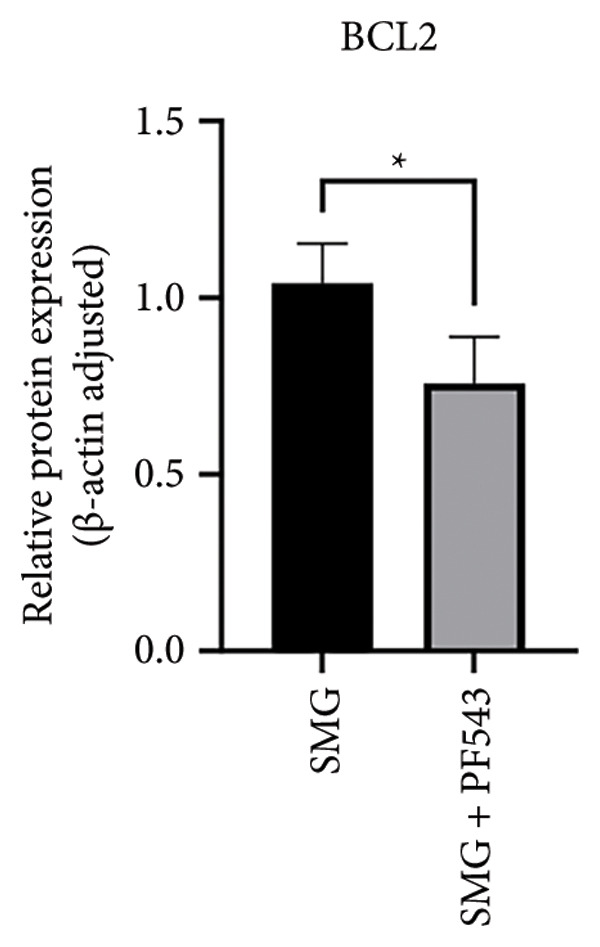
(c)

**Figure:**
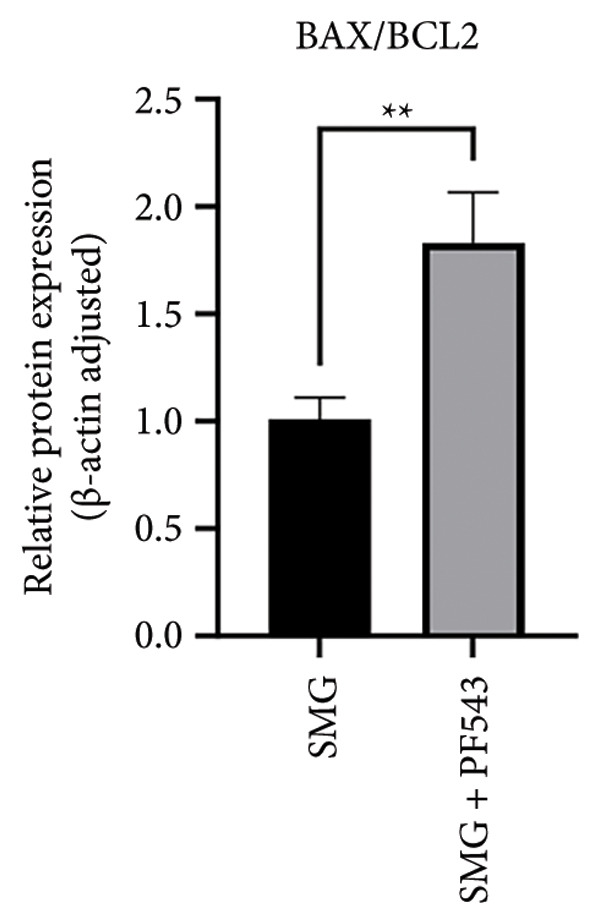
(d)

**Figure:**
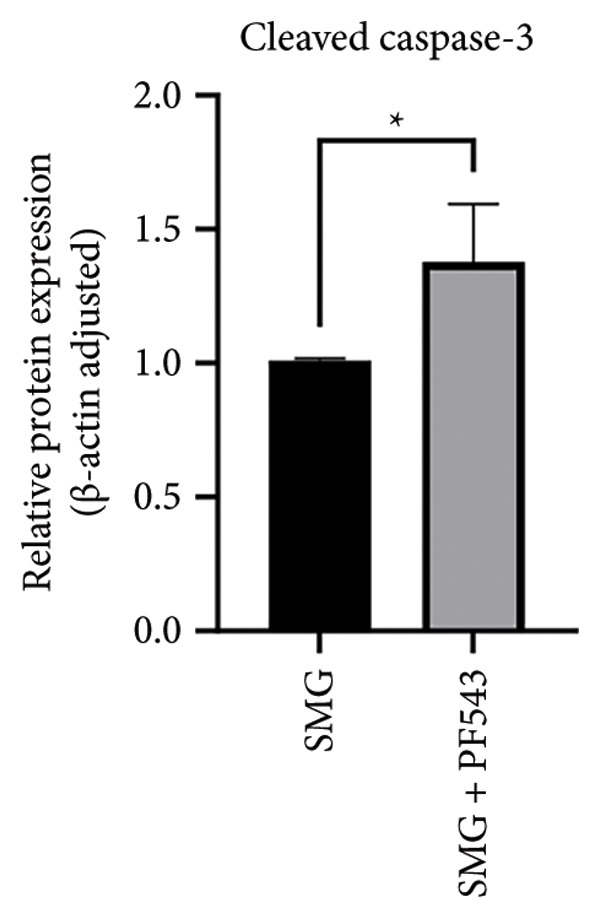
(e)

**Figure:**
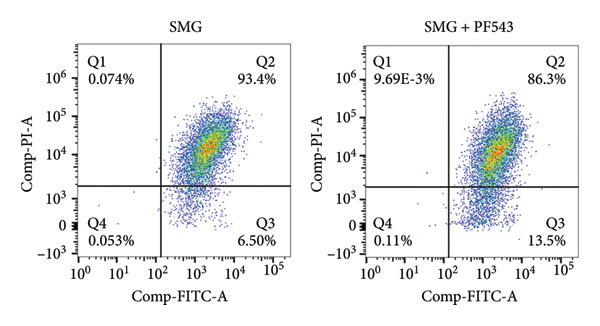
(f)

**Figure:**
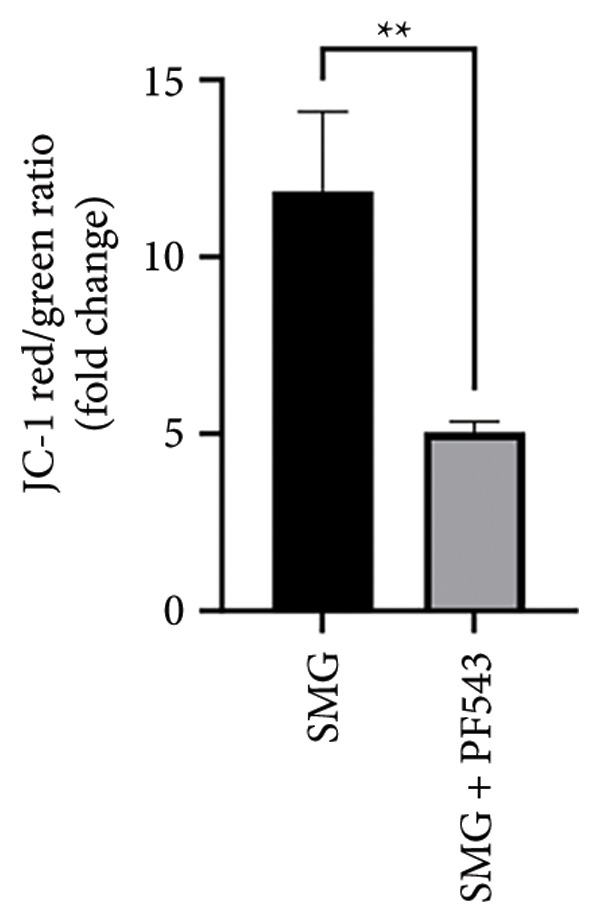
(g)

## 4. Discussion

Previous studies have shown that undifferentiated stem cells primarily generate energy through aerobic glycolysis, producing lactate as a major byproduct [23–25]. This metabolic strategy supplies the anabolic and catabolic substrates needed to sustain pluripotency. As stem cells undergo differentiation, mitochondrial biogenesis and maturation of oxidative metabolism appear to increase in parallel with the energy requirements of specialized cell functions [23, 24]. Supporting this, recent work demonstrated that cathepsin *k* deletion or inhibition enhances glycolysis in jaw bone marrow mesenchymal stem cells (JBMMSCs), thereby promoting alveolar bone regeneration [26]. These findings highlight the importance of glycolytic metabolism in regulating MSC behavior. Consistently, many studies have reported that MSCs rely predominantly on glycolysis for energy production in their undifferentiated state, shifting toward OXPHOS upon differentiation [27].

Earlier research from our group indicated that hDPSCs cultured under SMG conditions exhibit accelerated proliferation but reduced differentiation potential [28, 29]. This study expands on these observations by demonstrating for the first time that SMG enhances glycolytic activity in hDPSCs, a change likely linked to increased proliferation and reduced differentiation. This metabolic profile may facilitate improved cell survival, reduced apoptosis, and prolonged self‐renewal capacity under SMG conditions. Lactate, the principal product of glycolysis, has been shown to upregulate glycolytic gene expression under normoxic conditions, thereby enhancing MSC proliferation [12]. Undifferentiated stem cells are typically characterized by high glycolytic enzyme expression, increased lactate output, reduced mitochondrial DNA content, and immature mitochondrial morphology. This glycolytic bias limits mitochondrial reactive oxygen species (ROS) production, which preserves cellular integrity and supports the maintenance of stemness [30]. In contrast, differentiation into somatic lineages promotes ROS accumulation, potentially impairing differentiation capacity. The enhanced proliferation and preserved stemness of MSCs under SMG thus suggest a metabolic environment dominated by glycolysis.

The reduction of apoptosis that was observed under SMG conditions may be closely associated with increased glycolysis and elevated SphK1 expression. SphK1, a key sphingolipid kinase, modulates the balance between pro‐apoptotic (e.g., Bim) and antiapoptotic (e.g., BCL‐2) proteins, thereby promoting stem cell survival. Previous work demonstrated that SphK1 mediates NT‐3‐dependent activation of the cyclic AMP response element‐binding protein (CREB) in oligodendrocyte progenitor cells, upregulating BCL‐2 and downregulating Bim to enhance survival [31]. Furthermore, the SphK1/S1P receptor signaling network is recognized as a central regulator of stem cell proliferation, differentiation, and apoptosis through downstream activation of pathways, such as p42/44‐MAPK/ERK1/2 and PI3K/Akt [32–34].

The results of this study suggest that SMG markedly promotes glycolysis and suppresses apoptosis in hDPSCs. Proteomic profiling identified SphK1 as one of the upregulated proteins evident under SMG, suggesting that it may act as a key mediator of these effects. Because SphK1 is implicated in cell proliferation, differentiation, glycolysis, and apoptosis, its increased expression under SMG conditions is likely significant, prompting the hypothesis that it acts as a mediator of enhanced glycolytic activity and apoptotic suppression in hDPSCs in the context of SMG. Reports have shown that SphK1 can modulate glycolytic capacity through HIF‐1*α*, further linking SphK1 to metabolic regulation [19]. The SphK1/HIF‐1*α* signaling cascade has emerged as a central regulator of the anticancer effects of chrysin in hypoxia‐induced PC‐3 prostate cancer cells. Previous work demonstrated that chrysin administration markedly reduced the expression of SPHK1, HIF‐1*α*, and PARP, while at the same time triggering the cleavage of caspase‐3, a hallmark of apoptosis [35]. In a related context, the SphK1/S1PR3 axis has been shown to be upregulated in macrophages stimulated with lipopolysaccharide (LPS) as well as in the lungs of septic mice, where it drives the sequential activation of glycolysis‐promoting mediators, such as HIF‐1*α*, HK2, and PFKFB3. Pharmacological inhibition of SphK1 with PF‐543, a highly specific small‐molecule antagonist, effectively suppressed this axis both in vitro and in vivo and, importantly, alleviated sepsis‐related inflammation and multiorgan damage in animal models [30].

Building on these observations, this study employed PF‐543 to inhibit SphK1 in hDPSCs. This intervention led to a pronounced decrease in glycolysis‐associated markers, concomitant with elevated levels of apoptosis. Our data also demonstrate that hDPSCs cultured under SMG conditions exhibit enhanced proliferation and maintenance of stem cell characteristics, with glycolysis emerging as the predominant energy source. This pattern corroborates our earlier reports indicating that SMG augments glycolytic capacity in hDPSCs, which may underlie their increased proliferation, delayed differentiation, and preserved stemness. High levels of HIF‐1*α* have been implicated in promoting glycolytic metabolism while repressing OXPHOS activity in MSCs and thereby supporting an undifferentiated phenotype [36, 37]. Elevated mRNA expression and stabilization of hypoxia‐inducible factors appear to protect MSCs from apoptosis and contribute to the maintenance of their glycolytic profile. To further probe the role of glycolysis, we treated SMG‐cultured hDPSCs with the glycolytic inhibitor 2‐DG and observed increased expression of apoptosis‐related proteins alongside heightened apoptotic cell death.

Previous studies have also linked microgravity to apoptosis in various cell types. Lewis et al. first reported programmed cell death in cells grown under true microgravity conditions [38]. Using a rotating cell culture system (RCCS), researchers showed that human PBMCs exposed to SMG secreted significantly higher levels of TNF‐α, thereby enhancing apoptosis [39]. Comparable findings have been documented in lymphocytes, where random positioning machine (RPM)–induced SMG increased apoptotic cell death [40]. Additional evidence indicates that SMG can impair proliferation and differentiation while promoting apoptosis in bone marrow stem cells [41] and mouse embryonic osteoblasts [42]. In human osteoblasts, SMG exposure markedly elevated the Bax/BCL‐2 ratio, a classic indicator of apoptosis [43]. Interestingly, HUVECs proliferated more rapidly under SMG without concomitant apoptosis, an effect thought to be mediated by miR‐27b‐5p, which protects HUVECs from apoptotic stress [44]. Moreover, autophagy and mitophagy have recently been proposed as protective mechanisms mitigating endoplasmic reticulum stress–induced apoptosis in HUVECs exposed to SMG [45]. In contrast to the pro‐apoptotic responses frequently observed in other cell types, our data show that hDPSCs under SMG display reduced apoptosis. This cell type–specific outcome may reflect intrinsic differences in cellular metabolism, signaling networks, or microgravity simulation systems, highlighting the need for comparative studies across diverse stem cell populations. The distinctive response of hDPSCs to SMG suggests a unique adaptation of dental pulp stem cells that favors survival and stemness maintenance in low‐mechanical‐load environments.

The therapeutic potential of human MSCs is often limited by their restricted proliferative capacity and reduced viability during in vitro expansion, which constrains their widespread application in regenerative medicine. These findings provide the first evidence that SMG conditions can inhibit apoptosis in hDPSCs, potentially through increased SphK1 activity and enhanced glycolytic metabolism. This metabolic shift may contribute to the improved expansion and survival of hDPSCs, thereby offering a promising avenue to overcome the shortage of seed cells for tissue engineering. Collectively, our results not only illuminate the interplay between SphK1 signaling, glycolytic regulation, and cell survival under SMG but also underscore the potential of microgravity‐based culture systems to enhance the therapeutic utility of stem cells.

## Disclosure

All authors have approved the submitted version.

## Conflicts of Interest

The authors declare no conflicts of interest.

## Author Contributions

Jingyi Che and Zhengjun Qiu share equal contribution. Jingyi Che and Zhengjun Qiu performed the experiments and wrote the manuscript. Huailong Hou, Jingxuan Sun, Shuang Zhang, and Mengdi Li carried out the experiments and performed the data processing and statistical analysis. Weiwei Zhang designed the study. Yanping Li, Lina He, Shuang Pan, and Yumei Niu supervised the experiments. All authors contributed to the article. Jingyi Che and Zhengjun Qiu have contributed equally to this work and share first authorship.

## Funding

This research was funded by Key Research and Development Program of Heilongjiang Province, grant number 2022ZX06C05; Joint Guiding project of Heilongjiang Natural Science Foundation, grant number LH2022H043; and Young Medical Talent Training Funding Project of the First Affiliated Hospital of Harbin Medical University, grant number 2024YQ18.

## Data Availability

The data that support the findings of this study are available from the corresponding author upon reasonable request.
